# Comparison of the myocardial protective effect of sevoflurane versus propofol in patients undergoing heart valve replacement surgery with cardiopulmonary bypass

**DOI:** 10.1186/s12871-017-0326-2

**Published:** 2017-03-04

**Authors:** Xiao-Lin Yang, Dan Wang, Guo-Yuan Zhang, Xiao-Lan Guo

**Affiliations:** 1 Departments of Anaesthesiology, Affiliated Hospital of North Sichuan Medical College, 63# Wenhua Road, Shunqing District, Nanchong, Sichuan 637000 People’s Republic of China; 2 Laboratory Medicine, Affiliated Hospital of North Sichuan Medical College, Nanchong, Sichuan 637000 People’s Republic of China

**Keywords:** Myocardial protection, Cardiopulmonary bypass, Heart valve replacement, Sevoflurane, Propofol

## Abstract

**Background:**

This study aimed to compare myocardial protective effects of anaesthesia with intravenous infusion of propofol versus inhalation of sevoflurane in patients undergoing heart valve replacement surgery with cardiopulmonary bypass.

**Methods:**

Seventy-six patients undergoing valve replacement with cardiopulmonary bypass were randomly assigned to propofol or sevoflurane anesthesia during the surgery, respectively. For assessing myocardial injury, cardiac troponin I (cTnI) and creatine kinase isozyme (CK-MB) were determined before induction (T_0_), 0.5 h (T_1_) and 3 h (T_2_) after aortic unclamping, and 24 h (T_3_) and 48 h (T_4_) after surgery. The concentrations of interleukin (IL)-6 and IL-10 as the systemic inflammatory and anti-inflammatory markers were also measured at above time points.

**Results:**

In the sevoflurane group, the plasma concentrations of cTnI and CK-MB from T_l_ to T_4_ and the levels of IL-6 and IL-10 from T_1_ to T_2_ were lower than those in the propofol group. Moreover, a higher ratio of automatic heart beat recovery and a shorter length of intensive care unit or hospital stay were found in the sevoflurane group comparing with the propofol group.

**Conclusion:**

Sevoflurane anaesthesia produced more prominent myocardial protection and attenuated inflammatory response than propofol anaesthesia in patients with valve replacement surgery under cardiopulmonary bypass, resulting in shorter ICU and in-hospital stay.

**Retrospective clinical trial registration:**

Identified as ChiCTR-IOR-16009979 at http://www.chictr.org.cn/.

## Background

Cardiac surgical procedures unavoidably produce myocardial cell injury, which may originate from myocardial ischemia reperfusion, cardiopulmonary bypass (CPB), or operative procedure, etc. [1–5]. Previous studies suggested that both modern inhaled anesthetics (such as isoflurane or sevoflurane) and intravenous anesthetic propofol have the effect of myocardial preservation in different degree [6–9]. Nevertheless, most of those studies were performed in patients undergoing coronary artery bypass grafting (CABG), while few studies in non-CABG patients. Furthermore, high quality meta-analyses in adult cases have showed controversial or contradicting results [10–15]. In this study, we compared the cardioprotective effect of anesthesia with intravenous propofol and inhaled sevoflurane in patients undergoing mitral, aortic or tricuspid valve replacement surgery. Plasma cardiac troponin-I (cTnI) and creatine kinase isozyme (CK-MB) were used as the primary markers of myocardial cell injury, and the levels of interleukin (IL)-6 and IL-10 within 48 h after operation were used as the systemic inflammatory and anti-inflammatory markers.

## Methods

### Experimental protocol

This prospective, randomized control study was approved by our institutional ethics committee (Affiliated Hospital of North Sichuan Medical College, Nanchong, China). The written informed consents were obtained from 76 patients scheduled for open-heart cardiac surgery (uni-valve replacement of mitral, aortic or tricuspid valve due to stenosis or/and regurgitation) with cardiopulmonary bypass. All patients were American Society of Anaesthesiologists physical status II-IV, aged between 40 and 65 years, New York Heart Association classification of cardiac function II-III, ejection fraction greater than 40%, no history of nervous system diseases. The patients were randomly assigned to the sevoflurane group or the propofol group with equal size according to computer-generated randomization. The sevoflurane group and the propofol group were anaesthetized by inhaled sevoflurane or by infusion of propofol during the entire operative procedure, respectively. Exclusion criteria included hypertensive disease, coronary artery disease, diabetes, chronic obstructive pulmonary disease, infective endocarditis, hematogenic and immune systemic disease, perioperative steroid therapy, and contraindications for using propofol or sevoflurane.

### Primary and secondary end-points

The primary end-points were the changes of cTnI and CK-MB values from the beginning of anaesthesia to 48 h after operation. Secondary end-points included the changes of IL-6 and IL-10 during and within 48 h after operation, the ratio of automatic heart beat recovery, the requirements of intraoperative vasoactive agents, and short-term clinical outcomes (time of mechanical ventilation, length of ICU/hospital stay, awareness during operation by postoperative following up, and serious complications or death).

### Perioperative management

#### Anaesthetic technique

Patients were premedicated with intramuscular phenobarbital sodium 100 mg and anisodamine 10 mg 30 min preoperative. In the operating room, all patients were routinely monitored for the electrocardiogram, pulse oxygen saturation, end-tidal carbon dioxide pressure, invasive radial arterial pressure, central venous pressure and auditory evoked potential index (AEPi) with a PM-9000 express multifunctional monitor (Mindray Medical International Limited, Shenzhen, China). Transesophageal echocardiograph and urine output were also monitored. In the two groups, anaesthesia was induced with midazolam 0.1–0.2 mg kg^−1^ and fentanyl 10 μg kg^−1^. Vecuronium 0.15 mg kg^−1^ was given to facilitate tracheal intubation. Mechanical ventilation was controlled using 100% oxygen with a tidal volume of 6–8 ml kg^−1^. A normal end tidal carbon dioxide pressure (35–45 mmHg) was obtained by adjusted the respiratory frequency at 12–16 breaths/min. In the sevoflurane group, Anaesthesia was maintained by inhaled sevoflurane (1–5%), and fentanyl (5–10 μg kg^−1^) and vecuronium 0.1 mg kg^−1^ boli as needed. During CPB, sevoflurane was administered through the oxygenator. The depth of anaesthesia before, during and after CPB was controlled at AEPi 30–40, 15–30 and 30–40 by adjusted inhaled sevoflurane concentration, respectively [16–19]. In the propofol group, anaesthesia was maintained with propofol at a infusion rate of 3–10 mg · kg^−1^ · h^−1^, and fentanyl (5–10 μg kg^−1^) and vecuronium 0.1 mg kg^−1^ boli as needed; the depth of anaesthesia before, during and after CPB was also controlled at AEPi 30–40, 15–30 and 30–40 by adjusted the infusion rate of propofol, respectively.

#### Surgery and cardiopulmonary bypass

The cold cardioplegia solution was prepared by magnesium sulfate 2.5 g, potassium chloride 2 g, and sodium bicarbonate 0.5 g in 500 ml physiologic saline, and administered into the aortic root every 30 min. The CPB procedure and surgical techniques under moderate hypothermia (28–32 °C) were standardized. The two groups of patients were operated by the same group of cardiac surgeons. The CPB prime volume, 1000–1500 ml calculated by patient’s weight, contained lactate Ringer’s solution, hetastarch, mannitol, heparin and blood (depending on the expected pump haematocrit). The perfusion pressure (50–70 mmHg) was maintained by a continuous non-pulsatile blood-flow rate of 2.0–2.5 l min^−1^ m^−2^ during CPB. All patients were weaned off CPB by the support of small doses of dopamine and norepinephrine. The patients were withdrawn from the study if the CPB time was less 30 min or over 120 min or repeated CPB was more than two times.

#### Hemodynamic data

Global hemodynamic data (mean arterial pressure [MAP], central venous pressure [CVP], cardiac output) were recorded just before the start of surgery, before the start of CPB (pre-CPB), 15 min after the end of CPB, and at the end of the operation. Five consecutive beats were averaged.

#### Intensive care unit management

All patients were continuously supported by mechanical ventilation in the thoracic surgical intensive care unit (ICU) postoperatively. For analgesia and sedation, fentanyl 50–100 μg and midazolam 0.1 mg kg^−1^ were intravenous bolus according to clinical needs. Patients were extubated when they could maintain adequate spontaneous respiration and required minimal oxygen support. The amount of vasoactive drugs were recorded during operation and after admission to the ICU. Intraoperative awareness by operative questionnaire and the time of mechanical ventilation, as well as the length of ICU/hospital stay were documented.

#### Laboratory measurements

Blood samples (4 ml) were collected from the peripheral or central venous line at just before induction (T_0_), 30 min (T_1_) and 3 h (T_2_) after aortic unclamping, and 24 h (T_3_) and 48 h (T_4_) after operation. One ml of 4 ml blood sample was used to measure the hematocrit (Hct), and the rest 3 ml blood sample was used to measure the biomarkers of myocardial injury (cTnI, CK-MB), the inflammatory factor (IL-6) and anti-inflammatory factor (IL-10) in our hospital clinical chemistry department. The concentrations of cTnI and CK-MB were determined by immunochemistry analytic method (reagent kit provided by Abbott Lab, US), and the lower detection limit is 0.01 ng ml^−1^. IL-6 and IL-10 were measured using enzyme-linked immunosorbent assay (reagent kit provided by Rapid Bio, USA), and the detection thresholds were 9 pg ml^−1^ and 15 pg ml^−1^, respectively. To avoid the effect of hemodilution on experiment data, the following formula was used to calibration: theoretical value = measured value × (baseline Hct value/measured Hct value). The baseline Hct was measured before induction.

### Statistical analysis

A sample size of 70 cases would have a power of 95% to detect a difference in the level of cTnI of 2 ng ml^−1^ as a primary end-point using a two-sample *t*-test with 0.05 two-sided significance level [11, 20, 21]. Patient characteristics were compared with an unpaired *t* test and Fisher exact test where appropriate. Both biochemical serum markers and hemodynamic data were compared using a two-way analysis of variance techniques for repeated measurements in each group and between the two groups. All data were analyzed with the statistical package SPSS19.0 (SPSS Inc, Chicago, IL, USA) and expressed as mean (standard deviation) unless otherwise stated and statistical significance was accepted at *P* < 0.05.

## Results

**Fig. 1:**
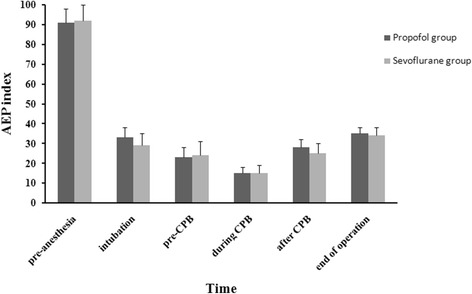
The changes of auditory evoked potential index (AEPi) in the two groups. The changes of AEPi were kept similar in pre-anaesthesia, intubation, pre-CPB, during CPB, after CPB and the end of operation between the two groups, respectively. CPB = Cardiopulmonary bypass

The study included a total of 76 patients undergoing valve replacement surgery with CPB. In the sevoflurane group, one patient was excluded from this study due to the CPB duration exceeding 120 min. In the propofol group, two patients were excluded from this study because one case needed repetitive CPB and the other one’s CPB duration exceeded 120 min. The two groups were comparable with respect to sex, age, weight, types of surgery, CPB time, aortic clamp time, arrest time, total administered dose of fentanyl (midazolam), and the incidence of intraoperative awareness. However, compared with the propofol group, less consuming doses of vasoactive drugs, a higher ratio of automatic heart beat recovery, a shorter length of ICU or hospital stay were found in the sevoflurane group (Table 1). The changes of AEPi in the two groups were kept similar (Fig. 1). Both mean arterial pressure and cardiac output after CPB were decreased in the propofol group but not in the sevoflurane group (Table 2). Central venous pressure was kept stable throughout in both groups. A similar trend of cTnI, CK-MB, IL-6 or IL-10 in the two groups was observed: the biomarkers increased very fast 30 min after aortic unclamping and peaked 3 h later, then started to decrease gradually. cTnI and CK-MB remained increased even at 48 h after operation, while IL-6 and IL-10 returned to baseline levels. However, the respective levels in the sevoflurane group were always lower than those in the propofol group in corresponding time points (Table 3).

**Table 1:** Patient characteristics

Patient characteristics	Propofol (*n* = 37)	Sevoflurane (*n* = 36)
Preoperative data		
Age (yr)	50.7 (6.6)	50.5 (6.4)
Weight (kg)	54.5 (7.9)	56.5 (11.8)
Sex (M/F)	18 / 19	16 / 20
ASA class	II–IV	II–IV
EF (%)	57.2 (5.6)	55.9 (5.4)
Types of surgery (n, %)		
Replacement of mitral valve	15 (40)	14 (39)
Replacement of aortic valve	9 (24)	10 (28)
Replacement of tricuspid valve	4 (10)	4 (11)
Replacement of mitral valve and shaping of tricuspid valve	9 (24)	8 (22)
Intraoperative data		
Operating time (min)	198 (28)	183 (34)
CPB time (min)	95 (18)	96 (17)
Aortic clamp time (min)	62 (21)	64 (18)
Ratio of automatic heart beat recovery (%)	62.2	83.3*
Incidence of intraoperative awareness (%)	0	0
Propofol (mg)	857.3 (166.5)	0
Sevoflurane (MAC hour)	0	4.6 (0.9)
Fentanyl (mg)	2.0 (0.2)	2.1 (0.3)
Midazolam (mg)	20.6 (2. 9)	22.8 (3.0)
Dopamine (mg)	57.3 (13.5)	45.9 (15.1)*
Nitroglycerin (μg)	130.3 (50.2)	102.2 (34.5)*
Epinephrine (μg)	148.7 (37.8)	111. 6 (19.6)*
Postoperative data		
Duration of mechanical ventilation time (hr)	9.4 (1.5)	6.2 (0.8)*
Serious complications or death (%)	0	0
ICU stay (hr)	48.6 (3.7)	42.3 (3.5)*
Hospital stay (d)	16 (13–19)	12 (9–15)*

Values are expressed as mean (SD) or median (range). *P < 0.05 *vs* the value of propofol group

*EF* Ejection fraction, *ASA class* American Society of Anaesthesiologists physical status classification, *MAC* minimum alveolar concentration, *MAC hr* inhaled anaesthetic concentration/MAC × inhaled time (hr)

**Table 2:** Perioperative Hemodynamic Data

Parameter	Start of surgery	Pre-CPB	Post-CPB	End of surgery
MAP (mmHg)				
Propofol	82 (3)	76 (5)	68 (4)*	71 (4)*
Sevoflurane	79 (4)	77 (5)	76 (3)^#^	80 (3)^#^
CVP (mmHg)				
Propofol	11 (3)	12 (2)	11 (3)	12 (3)
Sevoflurane	12 (3)	11 (3)	12 (2)	12 (2)
Cardiac output (l/min)				
Propofol	5.7 (0.7)	5.2 (0.8)	4.6 (0.7)*	4.7 (0.5)*
Sevoflurane	5.5 (0.8)	5.4 (0.9)	5.6 (0.6)^#^	5.4 (0.6)^#^

Data are mean (SD). * Different compared to before the start of surgery (*P* < 0.05). # Different between propofol and sevoflurane (*P* < 0.05)

*MAP*mean arterial pressure, *CVP* central venous pressure, *CPB* cardiopulmonary bypass

**Table 3:** Perioperative markers of myocardial injury and systemic inflammation

Marker	T_0_	T_1_	T_2_	T_3_	T_4_
cTnI (ng/ml)					
Propofol	0.01 (0.02)	18.26 (9.67)^#^	26.66 (9.10)^#^	13.71 (6.14)^#^	8.72 (4.83)^#^
Sevoflurane	0.02 (0.02)	10.17 (6.63)^#*^	15.17 (8.73)^#*^	9.00 (6.43)^#*^	4.62 (3.40)^#*^
CK-MB (u/l)					
Propofol	0.87 (0.50)	47.73 (9.30)^#^	86.12 (7.50)^#^	31.79 (6.29)^#^	14.35 (4.31)^#^
Sevoflurane	0.88 (0.60)	28.54 (7.69)^#*^	61.29 (8.80)^#*^	20.60 (7.84)^#*^	8.28 (2.16)^#*^
IL-6 (pg/ml)					
Propofol	15.86 (4.45)	36.89 (6.71)^#^	59.69 (7.53)^#^	25.80 (6.15)^#^	17.14 (5.17)
Sevoflurane	13.91 (5.00)	27.87 (7.83)^#*^	42.67 (8.89)^#*^	22.64 (6.92)^#^	15.44 (4.54)
IL-10 (pg/ml)					
Propofol	19.07 (4.75)	56.72 (8.36)^#^	73.73 (7.44)^#^	28.35 (6.90)^#^	20.58 (7.56)
Sevoflurane	16.79 (4.74)	38.24 (7.90)^#*^	54.29 (8.08)^#*^	23.70 (6.51)^#^	18.88 (5.50)

Data are given as mean (SD). T_0_ = before induction, T_1_ = 30 min after aortic unclamping, T_2_ = 3 h after aortic unclamping, T_3_ = 24 h after surgery, T_4_ = 48 h after surgery. ^#^ *P* < 0.01 vs. T_0_; ^*^ *P* < 0.05 vs. the corresponding value in the propofol group

## Discussion

Our study shows that patients receiving sevoflurane anaesthesia have better myocardial protective effect than patients receiving propofol anaesthesia for the heart valve replacement surgery under CPB. In the present study, both the cTnI and CK-MB were used as the sensitive and specific indicators of myocardial cell injury [22–25]. In order to meet the needs of a better comparability of cardioprotection between the two groups, a similar depth of anaesthesia was monitored by AEP index through adjusting the inhaled sevoflurane concentration or the infusion rate of propofol in different periods of surgical procedure (pre-, during and post-bypass) (Fig. 1), and the proportions of valve replacement in both groups are also similar (Table 1). Although surgical procedure itself - as heart valve replacement implicates direct myocardial injury and the leak of cTnI and CK-MB, a lower level of cTnI or CK-MB was found in the sevoflurane group. It implicates sevoflurane may alleviate the myocardial cell injury to some degree. Our present results are well coincident with previous studies by using inhaled anaesthetics in CABG or paediatric cardiac surgery [1, 2, 4, 20, 26, 27]. Studies have reported that the cardioprotection mechanism of inhaled anesthetics such as, volatile anesthetics open intracellular K_ATP_ channels, activate adenosine receptors, and inhibit Na^+^/K^+^ pump [1, 8, 28, 29]. Studies have also reported that the cardioprotection mechanism of propofol is related to its anti-inflammatory, immunomodulatory and antioxidant properties [21, 30, 31]. Nevertheless, many studies, such as some prospective randomized controlled trials [10–13, 32] and observational study [14] in adult patients indicated some contradicting results on the cardioprotection between volatile and non volatile agents. We speculate the reasons of these contradictory results may be related to the differences of patients’ conditions, anaesthesia protocols, surgery types and procedures, etc. For example, volatile anaesthetics or propofol were administered in any combination of the pre-, during and post-bypass period in previous studies. In Bignami’s study, no difference was found in the myocardial protective effect between sevoflurane and propofol [32], which may partly due to no administration of sevoflurane during CPB in the inhalation anesthesia group. On the contrary, in our present study, both sevoflurane and propofol were administered throughout the operative procedure, which may provide an optimal cardioprotection. In addition, the cardioprotective effect produced by sevoflurane or propofol may related to the concentration used in some degree. Laboratory investigations reported 1.0 MAC of volatile anaesthetics provides beneficial effect to cardiac injury. Lower concentrations of less than 0.75 MAC often showed no effect, whereas higher concentrations of more than 1.5 MAC did not result in further protective effect [1, 33, 34]. In this study, AEPi was used to conduct the regulation of inhaled sevoflurane concentration or the infusion rate of propofol in different surgical procedures. No intraoperative awareness occurred in the two groups implied that the administered sevoflurane concentration (1–5%) and the infusion rate of propofol (3–8 mg kg^−1^ h^−1^) were reasonable. However, the use of sevoflurane was associated with a significant increase in the ratio of automatic heart beat recovery after aorta unclamping, a more stable mean arterial pressure (Table 2) and a less inotropic support (Table 1) during operation. It indicated the depression of cardiac function in the sevoflurane group was less than that in the propofol group. In addition, that a significant reduction in the time of mechanical ventilation, length of ICU stay and the time to hospital discharge can make patients with valve replacement benefit from the use of sevoflurane throughout the entire procedure.

It is well-known that CPB and operative procedure often evoke a nonseptic systemic inflammatory reaction, with the potential risk of postoperative cardiac dysfunction [29, 30, 35]. The anti-inflammatory potential of sevoflurane has been reported in lots of CABG surgeries under CPB [36, 37]. When compared with the systemic delivery of sevoflurane, Kortekaas and co-authors in their study found that the intramyocardial delivery of sevoflurane can more strongly attenuate the systemic inflammatory response after CPB [38]. The results of this study also indicated sevoflurane possesses a strong property to depress the systemic inflammatory response in patients with valve replacement surgery. In this study, the lower plasma levels of prime proinflammatory cytokine IL-6 and anti-inflammatory cytokine IL-10 in the sevoflurane group during 48 h after surgery, indicates that sevoflurane anaesthesia attenuated postoperative cytokine response vs. propofol anaesthesia. This effect, on top of sevoflurane cardiac protective effect, may have independently contributed to the enhanced recovery of the patients.

Our study has several limitations. Firstly, although the patients could be randomly assigned to the sevoflurane group and the propofol group, the anaesthesiologists could not be blinded to the anaesthetic technique used in each group. However, the experimenters who collected the postoperative data and the laboratory staff were blinded to the randomization. Secondly, the data resulted from a relatively small number of patients. Therefore, more patients were needed for further study. Thirdly, in some degree, our present study lacked a clinically important positive outcome, which may due to a less myocardial ischemia or injury in non-CABG cases than that in CABG cases, or a effective myocardial protection either produced by sevoflurane or propofol. Another limitation was that a relatively short CPB time was selected. However, the cardioprotective effect of sevoflurane or propofol in those patients with multi-valve replacement or longer CPB time needs a further multi-center clinical study.

## Conclusions

In conclusion, patients receiving sevoflurane for uni-valve replacement surgery under CPB had significantly lower postoperative release of cTnI or CK-MB, and lighter inflammatory response than patients receiving propofol for the same procedure. Our findings suggest the administration of inhaled sevoflurane throughout the entire procedure can produce more significant myocardial protection and resulted in shorter ICU and in-hospital stay which may have further economic implications than the intravenous infusion of propofol.

## Abbreviations

AEPi: Auditory evoked potential index
ASA: American Society of Anaesthesiologists
CABG: Coronary artery bypass grafting
CPB: Cardiopulmonary bypass
cTnI: Cardiac troponin I
Hct: Hematocrit
ICU: Intensive care unit
IL: Interleukin
